# Engineering of Stimulus-Responsive Pirfenidone Liposomes for Pulmonary Delivery During Treatment of Idiopathic Pulmonary Fibrosis

**DOI:** 10.3389/fphar.2022.882678

**Published:** 2022-04-25

**Authors:** Meishan Han, Yingjian Song, Sha Liu, Xiaoyan Lu, Linyu Su, Meixuan Liu, Xiaosu Zhu, Kaoxiang Sun, Yanan Lu, Aiping Wang

**Affiliations:** ^1^ School of Pharmacy, Key Laboratory of Molecular Pharmacology and Drug Evaluation, Ministry of Education, Collaborative Innovation Center of Advanced Drug Delivery System and Biotech Drugs in Universities of Shandong, Yantai University, Yantai, China; ^2^ Department of Thoracic Surgery, The Affiliated Yantai Yuhuangding Hospital of Qingdao University, Yantai, China

**Keywords:** pirfenidone, idiopathic pulmonary fibrosis, stimulus-responsive liposomes, pulmonary administration, permeability of the mucus layer

## Abstract

Idiopathic pulmonary fibrosis (IPF) is an interstitial lung disease characterized by progressive and irreversible loss of lung function. Clinically safe and efficacious drug treatments for IPF are lacking. Pirfenidone (an anti-inflammatory, antioxidant and anti-fibrotic small-molecule drug) is considered a promising treatment for IPF. Unfortunately, several disadvantages of pirfenidone caused by traditional administration (e.g., gastrointestinal reactions, short elimination half-life) hinder its implementation. We designed pirfenidone pH-sensitive liposomes (PSLs) to target the acidic microenvironment of IPF and act directly at the disease site through pulmonary administration. Pirfenidone was encapsulated in liposomes to extend its half-life, and modified with polyethylene glycol on the surface of liposomes to improve the permeability of the mucus layer in airways. *In vitro*, the cytotoxicity of pirfenidone PSLs to pulmonary fibroblasts was increased significantly at 48 h compared with that using pirfenidone. In a murine and rat model of bleomycin-induced pulmonary fibrosis, pirfenidone PSLs inhibited IPF development and increased PSL accumulation in the lungs compared with that using pirfenidone solution or phosphate-buffered saline. Pirfenidone PSLs had potentially fewer side effects and stronger lung targeting. These results suggest that pirfenidone PSLs are promising preparations for IPF treatment.

## 1 Introduction

Idiopathic pulmonary fibrosis (IPF) is a progressive interstitial lung disease with clinical symptoms such as dyspnea, dry cough, and decreased lung volume (Richeldi et al., 2017). In the United States, the incidence of IPF in individuals over 65 years is 495.5 cases per 100,000 people (Raghu et al., 2013). The incidence is increasing, and IPF is controlled ineffectively due to its unknown pathogenesis (Kreuter et al., 2015; de Brito et al., 2020). The 5-years survival of IPF patients has been reported to be lower than that of many cancers (Schwartz et al., 1994; Bjoraker et al., 1998; King et al., 2001). Lung transplantation is the only clinically proven effective treatment strategy. Unfortunately, lung transplantation has resulted in poor tolerance by patients due to high treatment costs, lack of matched donors, and rejection reactions after surgery (Raghu et al., 2011).

Pirfenidone is a synthetic pyridone compound. It was approved for IPF treatment by the US Food and Drug Administration in 2014 and has been used worldwide (Schaefer et al., 2011; Rafii et al., 2013; Raghu et al., 2013; Varone et al., 2018). Notably, pirfenidone has been reported as a potential candidate for the treatment of COVID-19 (Seifirad, 2020). Pirfenidone has been shown to down-regulate the expression of cytokines and ACE receptors through a variety of mechanisms to reduce inflammation (Papakonstantinou et al., 2016; Li et al., 2017; Fois et al., 20182018). However, severe gastrointestinal reactions caused by oral administration have undermined the quality of life of patients (Rasooli et al., 2018). Recently, pulmonary inhalation administration has been applied to the local delivery of pirfenidone (Sakamoto et al., 2021). Compared with traditional oral administration, the pulmonary drug delivery system (PDDS) is a potential strategy due to the high effective dose and prolonged residence time in the lungs (da Silva et al., 2017). Nevertheless, the inhalation route must overcome the obstruction of the airway mucus layer to the drug particles (Porsio et al., 2018). Moreover, the abundant capillaries in the lungs allow unwanted drugs to enter the systemic circulation, increasing the potential risk of systemic adverse reactions. Also, multiple administrations caused by the short elimination half-life are not conducive to expanding the clinical application of pirfenidone. Therefore, the development of a targeted, high-efficiency, and safe PDDS is needed urgently in clinical practice to reduce the occurrence of adverse reactions and potential risks in drug-dose control.

Liposomes have been studied in depth due to their biocompatibility and high encapsulation efficiency (EE), and approved for marketing (Anselmo and Mitragotri, 2016; Prasad et al., 2015). The advantages of liposomes in drug delivery to the pulmonary system include low levels of irritation, and suitable particle sizes (∼100 nm) have been reported and studied widely (Cryan, 2005; Bulbake et al., 2017). Simultaneously, polyethylene glycol (PEG) modifies the surface to make liposomes hydrophilic and electrically neutral, thereby increasing the permeability of the mucus layer of airways to achieve efficient pulmonary drug delivery (Lai et al., 2007; Xu et al., 2020; Rommasi and Esfandiari, 2021).

In addition, the acidic microenvironment of IPF caused by long-term chronic inflammation provides the basis for the design of lung-targeted responsive drug delivery systems (DDS) (LozoVukovac et al., 2014). Therefore, we developed PEGylated pH-sensitive liposomes (PSLs) for delivery to the pulmonary system to overcome mucus in airways, promote lung targeting, reduce the adverse effects of pirfenidone, as well as extend the duration of action. The rate of mucus penetration of the preparation was measured using artificial sputum medium (ASM) to simulate human lung mucus. *In vitro* studies were undertaken to study the cytotoxicity of formulations using human embryonic lung fibroblasts. The advantages of administration to the pulmonary system were evaluated by pharmacokinetic studies in rats suffering from pulmonary fibrosis. The results from intravital imaging studies in mice suffering from pulmonary fibrosis were compared with those based on intravenous injection. A biodistribution study and the anti-fibrosis effects of formulations in rats with pulmonary fibrosis were studied through intratracheal administration of PSLs.

## 2 Materials and Methods

### 2.1 Materials and Animals

Pirfenidone (purity = 99%) was purchased from Shanghai Shifeng Biotech (Shanghai, China). 1,2-Dioleoyl-sn-glycero-3-phospho-ethanolamine (DOPE) was obtained from AVT Pharmaceuticals (Shanghai, China). The lipid 1,2-distearoyl-sn-glycero-3-phosphoethanolamine-N-[methoxy (polyethylenglycol)-2000] (DSPE-PEG2000) was purchased from Shanghai Ponsure Biotech (Shanghai, China). Cholesteryl hemisuccinate (CHEMS) was obtained from Sigma–Aldrich (Saint Louis, MO, United States). Ammonium sulfate was purchased from Zhiyuan Chemical Reagents (Tianjin, China). Sodium chloride and sodium hydroxide were obtained from Hengxing Chemical Reagent Manufacturing (Tianjin, China). Phosphate-buffered saline (PBS; pH 7.4 and pH 5.5) was from HyClone (Logan, UT, United States). Pig-stomach mucosa, deoxyribonucleotide sodium salt (DNA) from salmon, compound amino acid powder, and diethylene triamine pentaacetic acid (DTPA) were obtained from Sigma–Aldrich.

The human embryonic lung fibroblast cell line WI-38 was provided by Beina Biotechnology (Beijing, China). Male Sprague–Dawley rats (200 ± 20 g) and male Institute of Cancer Research (ICR) mice (25 ± 5 g) were purchased from Pengyue Experimental Animal Breeding (Jinan, China). Animal experiments were carried out in accordance with the UK Animals (Scientific Procedures) Act, 1986, and associated guidelines, and the EU Directive 2010/63/EU for animal experiments.

### 2.2 Preparation of Pirfenidone PSLs

The ammonium sulfate gradient method was modified and employed to prepare pirfenidone PSLs to improve the EE of the drug (Lu et al., 2019). Briefly, DOPE, CHEMS and DSPE-PEG2000 were dissolved in an appropriate volume of absolute ethanol, and then evaporated into a lipid film in a water bath at 50°C under at atmosphere of nitrogen. Ammonium sulfate solution (250 mmolL^−1^) was adjusted to an alkaline solution using NaOH solution. Under vigorous shaking, the lipid membrane was hydrated by the pre-prepared alkaline solution, which resulted in a suspension of blank PSLs. A micro liposome extruder coupled with a 100-nm polycarbonate membrane was applied 13-times to the suspension to prepare small unilamellar vesicles (SUVs). After overnight dialysis, the SUVs were incubated with pirfenidone at 55°C for 15 min to obtain PSLs. The latter were eluted thrice using PBS through ultrafiltration (2,500 × *g* for 40 min at room temperature) and stored at 4°C until use.

PSL unmodified with PEG were prepared by the same method as above, the only difference being that DSPE-PEG2000 was not used in the preparation material.

### 2.3 Characterization of Pirfenidone PSLs

Transmission electron microscopy using a JEM-1400 system (JEOL, Tokyo, Japan) was carried out to evaluate PSL morphology. The liposome solution was dripped onto a copper mesh with a carbon support film and dried naturally. Then, a 2% phosphotungstic acid solution (pH 7.4) was dripped onto the copper mesh to stain for imaging. The particle size and zeta potential were measured by dynamic light scattering using a Delsa Nano Series. The liposome solution was diluted with deionized water to ensure an appropriate scattering intensity. The EE of pirfenidone PSLs was calculated using the following formula:

$$\%EE=\frac{Weight\;of\;drug\;in\;purified\;liposomes}{Weight\;of\;drug\;in\;unpurified\;liposomes}\times 100\%$$

Briefly, purified liposomes and unpurified liposomes were diluted with methanol and vortex-mixed vigorously for 3 min. The absorbance of pirfenidone was measured using an ultraviolet spectrophotometer (UV-2450; Shimadzu, Kyoto, Japan), and the pirfenidone concentration was calculated. These measurements were done in triplicate and the results presented as the mean ± standard deviation (SD).

The *in vitro* release of PSLs in PBS at pH 7.4 or 5.5 was studied to evaluate the sensitivity of liposomes to acidic conditions. Briefly, 1 ml of a PSL solution or pirfenidone solution was added to a dialysis bag. This was followed by incubation in 50 ml of release media with different pH values at 37°C and continuous agitation at 50 rpm. The release medium (1 ml) was removed at 0.083, 0.5, 1, 2, 4, 6, 8, 12, 24, and 48 h to determine pirfenidone release, and the same volume of fresh release medium was replenished. The absorbance of the sample at 317 nm was measured by the ultraviolet spectrophotometer. Cumulative release was calculated as the percentage of the amount of drug in the release medium in comparison with the total amount of drug added at the beginning of the experiment.

### 2.4 *In Vitro* Studies

#### 2.4.1 ASM Penetration

ASM was prepared according to the method described by Sriramulu and others (Sriramulu et al., 2005). Type-II pig-stomach mucosa, DNA, DTPA, NaCl, KCl, and compound amino acid powder were dissolved in 50 ml of sterile ultrapure water with 250 μL of sterile egg-yolk emulsion. Then, the sterile NaOH solution was used to adjust the pH to 7.0. The prepared ASM was stored at 4°C until use.

ASM (30 μL) and PBS (1,000 μL) were added, respectively, to the donor side and recipient side of a Transwell™ chamber (12 well). After shaking for 15 min, 200 μL of PSL, or PSL with unmodified PEG or free-pirfenidone (1 mgmL^−1^) was added dropwise into the donor side. A 100-µL aliquot was removed from each recipient side at 0.5 and 1 h, mixed with methanol to destroy liposomes, and vortex-mixed vigorously for 3 min. The absorbance of pirfenidone was measured using the ultraviolet spectrophotometer and the concentration was calculated. The apparent permeability coefficient (P_app_) was calculated using the following equation:

$$P_{app=}\frac{dQ}{dt}\times \frac{1}{A\times C_{0}}$$

where dQ/dt is the amount of drug transferred from the donor chamber to the recipient chamber, C_0_ is the initial concentration of pirfenidone in the donor chamber, and A is the area (cm^2^) of the filter membrane.

#### 2.4.2 Study of Growth Inhibition

Human embryonic lung fibroblasts (WI-38 cells) were cultured in special medium for WI-38 cells at 37°C in an atmosphere of 5% CO_2_.

Inhibition of cell proliferation of the PSL solution and pirfenidone solution was studied by the 3-(4,5-dimethylthiazol-2-yl)-2,5-diphenyltetrazolium bromide (MTT) assay. WI-38 cells were seeded and cultured into 96-well plates at 5,000 cells per well. After 24 h, 100 μL medium containing different concentrations of PSL solution and pirfenidone solution was added into 96-well plates to replace the culture medium. Respectively, samples were incubated for 24 h or 48 h, 20 μL of MTT solution (5 mgmL^−1^) was added to each well, and incubation allowed for 4 h. After the cells had been washed thrice with PBS, 200 μL of dimethyl sulfoxide solution was added to dissolve formazan crystals. The absorbance of the cells was read using a multi-function microplate reader (Cytation5; BioTek, Winooski, VT, United States) at 570 nm.

Cell viability was calculated as the ratio of the absorbance of treated cells to that of untreated cells. Results were expressed as the half-maximal inhibitory concentration (IC_50_) of pirfenidone to cell growth compared with that in untreated cells. The cell-proliferation inhibition of blank PSLs was also studied using the same method.

### 2.5 *In Vivo* Studies

#### 2.5.1 Pharmacokinetic Study

The difference in pharmacokinetic distribution between pulmonary administration and intravenous administration was studied in rats with pulmonary fibrosis. A model of pulmonary fibrosis in Sprague–Dawley rats was established by intratracheal injection of bleomycin sulfate solution (6 mgmL^−1^,5 mgkg^−1^) using a liquid aerosol device (Shanghai Yuyan Instruments, Shanghai, China). Rats were divided randomly into four groups with six animals in each group. Two groups were administered 4 mgkg^−1^ of pirfenidone solution or PSLs *via* the tail vein. The other two groups were administered (i.t.) 4 mgkg^−1^ of pirfenidone solution or PSLs using a liquid aerosol device. Samples of whole blood were collected from the intraocular canthal venous plexus at 5 and 30 min, as well as 1, 2, 4, 6, 8, 12, 24, 48, and 72 h following administration. Plasma samples were centrifuged immediately at 2,500 × *g* for 10 min at 4°C and stored at −80°C until analyses.

#### 2.5.2 Biodistribution Study

Rats with pulmonary fibrosis were administered (i.t.) 4 mgkg^−1^ of pirfenidone solution or PSLs using a liquid aerosol device. Rats were sacrificed and tissues (heart, lung, spleen, liver and kidney) were isolated 0.5, 2, 4, and 24 h after administration. Each Gram of tissue sample was added to 3 ml of PBS before homogenization, and then centrifuged immediately at 13,500 × *g* for 5 min at 4°C to collect the supernatant. Supernatant samples were stored at −80°C until analyses.

#### 2.5.3 Analytical Conditions

Liquid chromatography–tandem mass spectrometry using the LC-30AD system and AB Sciex Triple Quad™ 4,500 setup (Sciex, Redwood City, CA, United States) was employed to analyze samples of plasma and tissue homogenates. Analyses were undertaken on a 3.5-µm Eclipse plus C_18_ (2.1 × 100 mm) column (Agilent Technologies, Santa Clara, CA, United States) at a flow rate of 0.5 mlmin^−1^ and column temperature of 35°C. The multi-reaction monitoring transition of pirfenidone was 186.0→93.0, and pirfenidone was separated by a mobile phase composed of water and acetonitrile (75:25, *v/v*).

#### 2.5.4 *In Vivo* Targeting Study

Mice with pulmonary fibrosis were administered DiR-PSL (0.25 mgkg^−1^) *via* tail-vein injection or intratracheal spray. A model of pulmonary fibrosis in ICR mice was established by intratracheal injection of bleomycin sulfate solution (2 mgmL^−1^,4 mgkg^−1^) using a liquid aerosol device. Mice were anesthetized and then photographed using a live imaging system (excitation wavelength: 710 nm; emission wavelength: 780 nm) at 1, 2, 4, 6, 8, and 10 h after administration. Finally, tissues (heart, lung, spleen, liver and kidney) were separated from sacrificed mice and images denoting fluorescence distribution were taken.

#### 2.5.5 Study of Anti-Fibrosis Efficacy

Twelve Sprague–Dawley rats suffering from pulmonary fibrosis were divided randomly into three groups. Rats in each group were administered 4 mgkg^−1^ of pirfenidone solution, PSLs, or PBS every 2 days for seven consecutive administrations. Rats were sacrificed after 14 days of treatment to obtain lung tissue and to prepare paraffin sections for staining [hematoxylin and eosin (H&E), Masson’s trichrome]. Each stained sample was observed at ×100 magnification by a fluorescence microscope (Olympus, Tokyo, Japan) and photographed.

### 2.6 Statistical Analyses

Data are the mean ± SD. One-way analysis of variance or Student’s t-test was used to indicate differences between two groups through Prism 6 (GraphPad, San Diego, CA, United States. *p* < 0.05 was considered significant.

## 3 Results and Discussion

### 3.1 Preparation of PSLs

**FIGURE 1:**
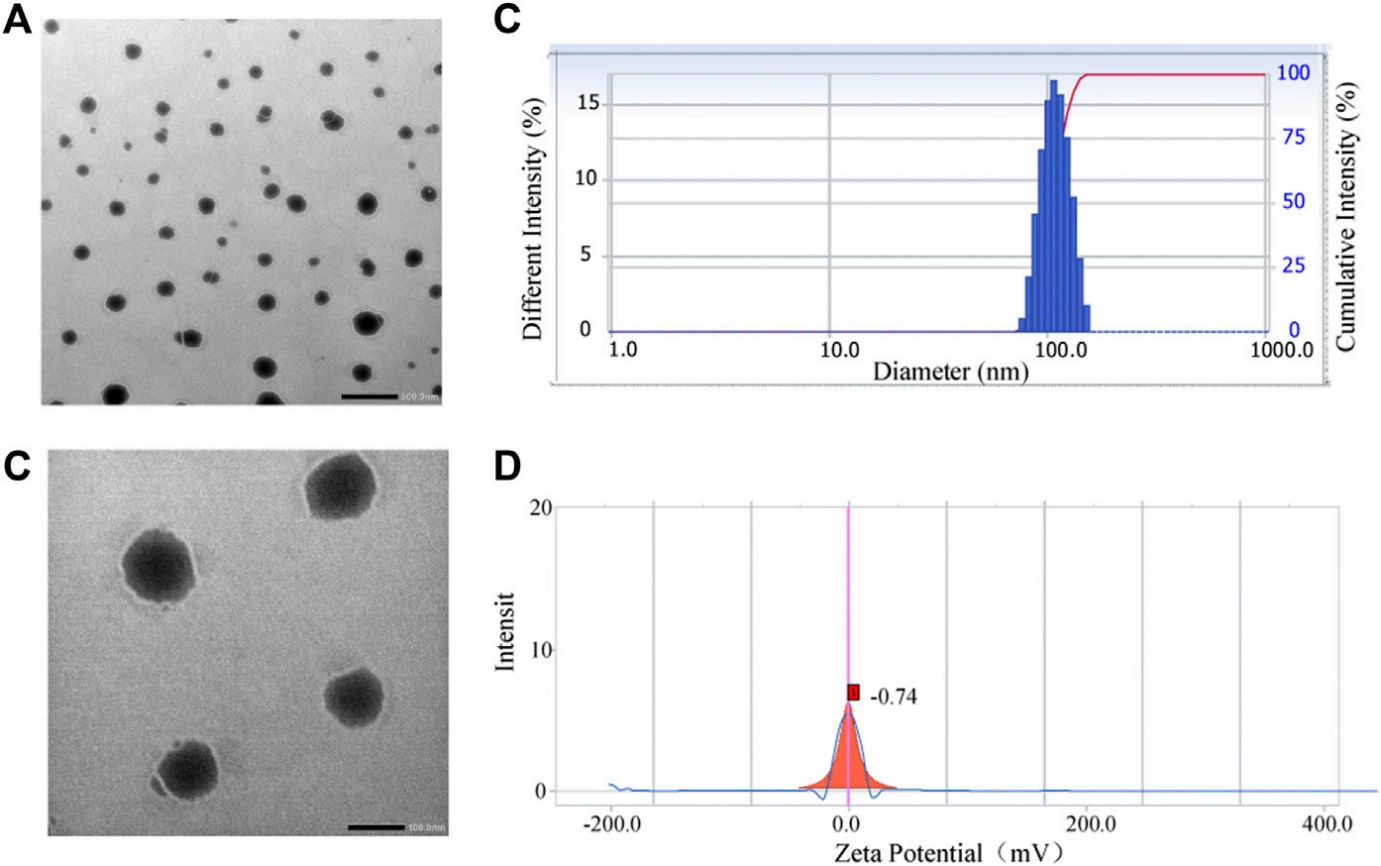
Characterization of PSLs. Typical TEM images of PSLs **(A,B)**, scale bar = 500 nm **(A)**, scale bar = 100 nm **(B)** Size distribution of PSLs **(C)** Potential distribution of PSL **(D)**.

PSLs with high EE were prepared by a slightly modified version of the ammonium sulfate gradient method. The calcium acetate gradient method should be the first choice for the loading of active drugs due to the weak acidic of pirfenidone. However, use of solutions of calcium acetate with different concentrations leads to incomplete hydration. In view of the low EE using passive drug loading, the ammonium sulfate solution was adjusted to be alkaline and employed to hydrate and increase the EE. The EE of pirfenidone liposomes prepared by the modified ammonium sulfate gradient method could be increased to 86.65 ± 1.36%. The morphology of PSLs was observed under a transmission electron microscope to be quasi-spherical (Figures 1A,B). The mean particle size of PSLs was 106.6 ± 0.35 nm, and the polydispersity index of PSLs was 0.061 ± 0.001. The distribution of particle sizes was relatively uniform. (Figure 1C). It has been reported that a particle size of ∼100 nm is beneficial for drug delivery to the pulmonary system, and increases the residence time in the lungs (Ghumman et al., 2021). The potential of PSLs was measured to be −0.74 mV (Figure 1D), which is close to neutrality. Hence, the PEG modified on the liposome surface reached sufficient density to penetrate the mucus layer (Wang et al., 2008). At the same time, the particle size and potential of blank liposomes were also measured using the same method. The particle size of the blank liposome was 104.2 ± 2.3 nm, the PDI was 0.060 ± 0.001, and the potential was −0.23 mv. The results showed that the particle size and potential of liposomes did not change significantly after drug loading.

**FIGURE 2:**
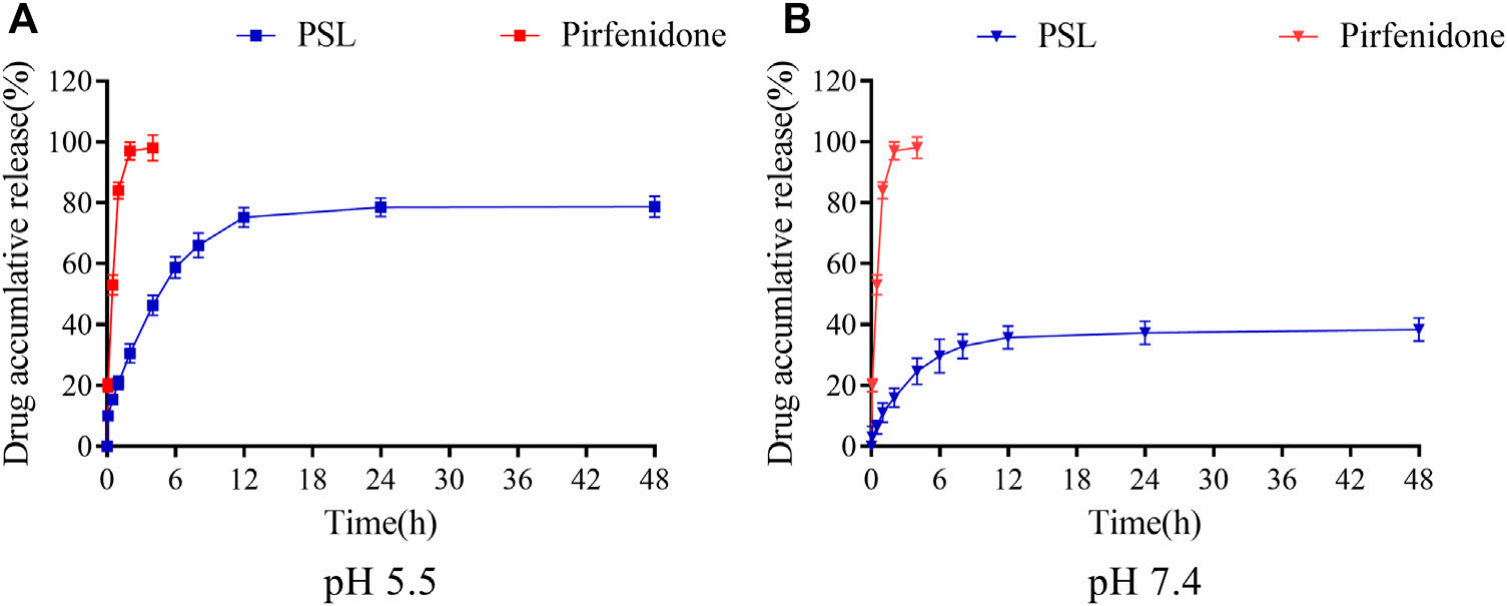
*In vitro* release curve of PSLs and pirfenidone in PBS of pH 5.5 **(A)** and pH 7.4 **(B)**.

The pH of the microenvironment in people with IPF has been reported to be ∼5.5 (LozoVukovac et al., 2014). Therefore, the release of PSLs in PBS at pH 5.5 and 7.4 was studied. PSLs were released effectively at pH 5.5 compared with that at pH 7.4 (Figure 2), and 80% of the encapsulated drug was released during 24 h. The rapid release in acidic PBS showed that the PSLs we developed had obvious pH responsiveness, and could achieve rapid release of a drug in an acidic microenvironment. Simultaneously, compared with the rapid release of pirfenidone solution, PSLs had long-lasting release, indicating that the action time of pirfenidone could be prolonged by PSLs.

### 3.2 ASM Penetration

**FIGURE 3:**
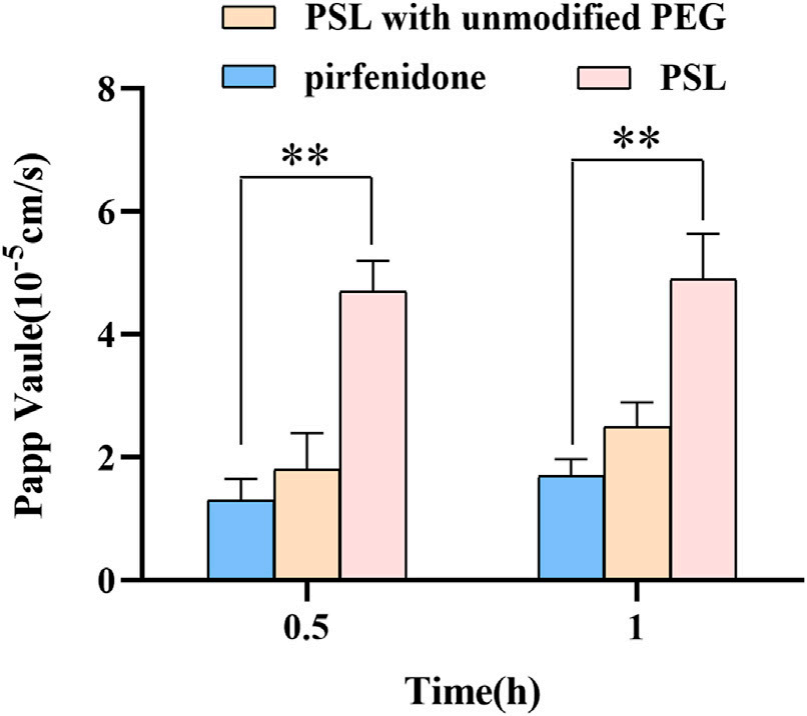
P_app_ of PSLs, PSLs with unmodified PEG, and pirfenidone during incubation with artificial sputum medium (P_app_: The apparent permeability coefficient. PSL unmodified with PEG: Pirfenidone pH-sensitive liposomes without surface modification with PEG 2000; **p < 0.01, *n* = 3).

ASM has been shown to have similar properties to that of the mucus layer in the human trachea (Sriramulu et al., 2005). ASM can be used to predict the penetration ability of a preparation in the mucus layer of airways. The mucus permeability of PSL was 2.6-times higher than that of PSLs with unmodified PEG, and 3.6-times higher than that of the free-pirfenidone solution (Figure 3). This result indicates that the mucus-penetration ability of the formulation was improved significantly by modifying the surface of liposomes with PEG. Therefore, the amount of drug entering the lungs after pulmonary administration was also increased significantly. This phenomenon could be because PEG-modified PSLs are hydrophilic and electrically neutral, and can penetrate the mucus layer of airways with the same characteristics as mucus (Wang et al., 2008; Popov et al., 2016). However, the mucus layer cannot be penetrated by PEG of any molecular weight. Studies have shown that low-molecular-weight (e.g., 2 kDa) PEG is suitable for penetrating the mucus layer. Conversely, high-molecular-weight PEG causes the carrier to adhere to the mucus layer and be removed (Wang et al., 2008). Simultaneously, the density of PEG on the surface of the carrier will also have a great impact upon penetration. A low density of PEG will mean that the carrier cannot achieve electrical neutrality; −7 mV is a critical point. If the carrier potential is less than the critical point, it indicates that the PEG density of the surface modification is insufficient, and the penetration rate will be reduced greatly (Wang et al., 2008). Therefore, using a certain density of low-molecular-weight PEG to modify the carrier was an effective strategy to penetrate the mucus layer of the lung.

### 3.3 Study to Measure Growth Inhibition

**FIGURE 4:**
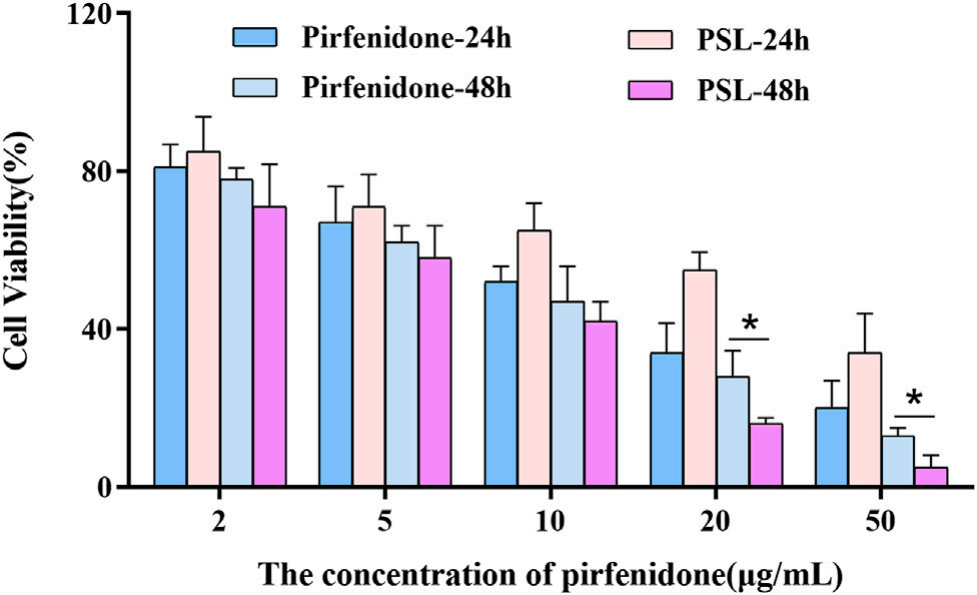
Influence of PSLs and pirfenidone on the viability of WI-38 cells after 24 and 48 h (WI-38 cells: The human embryonic lung fibroblast cell line. *p < 0.05, *n* = 6).

**FIGURE 5:**
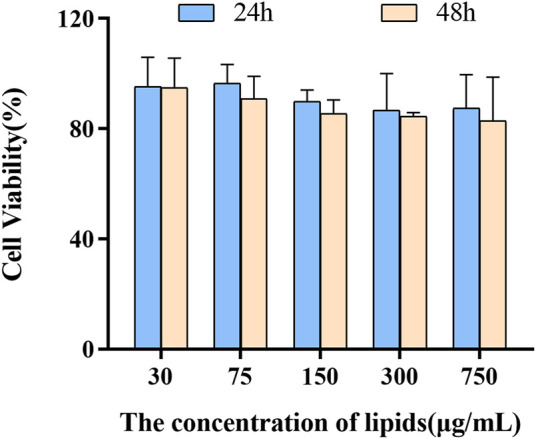
Influence of blank PSLs on the viability of WI-38 cells after 24 and 48 h (blank PSLs: pH-sensitive liposomes without pirfenidone, WI-38 cells: The human embryonic lung fibroblast cell line. *n* = 3).

The cell proliferation inhibition of PSLs compared with that of pirfenidone solution was dependent upon concentration and time (Figure 4). Twenty-four hours after drug treatment, PSLs did not show advantages compared with pirfenidone solution, possibly due to their sustained release. Forty-eight hours after sustained drug release, the viability of WI38 cells began to decrease, and cell proliferation inhibition ability was enhanced significantly compared with that of pirfenidone solution. These results indicated that the pirfenidone dose could be decreased by PSLs to reduce the risk of adverse reactions. In the cytotoxicity determination of blank PSLs, the materials used to prepare PSLs were considered to be non-toxic to cells and did not interfere with the cytotoxicity results of the formulations (Figure 5).

### 3.4 *In Vivo* Studies

#### 3.4.1 Pharmacokinetic Study

**FIGURE 6:**
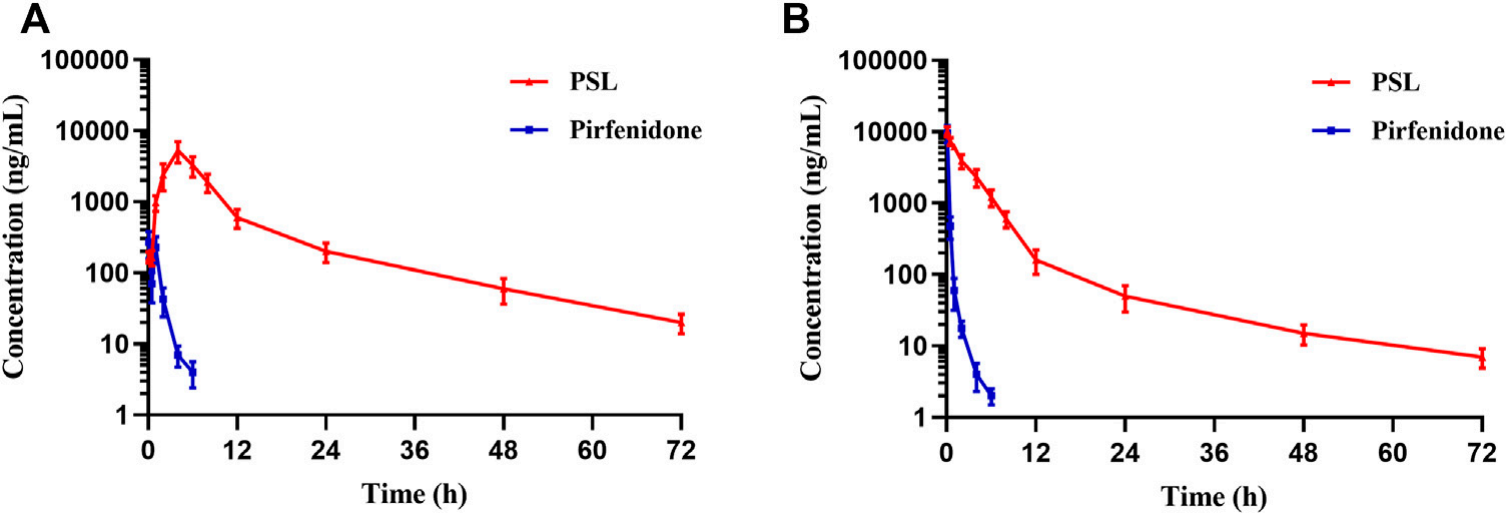
Mean plasma concentration–time curve of pulmonary administration **(A)** and intravenous injection **(B)** of PSLs and pirfenidone in a model of pulmonary fibrosis in rats (4 mgkg^−1^) (*n* = 6).

To evaluate the *in vivo* behavior of PSLs and compare the difference between intravenous injection and pulmonary administration, plasma samples from rats that had experienced different administration methods at different time points were collected and measured. The mean plasma concentration–time profiles are presented in Figure 6, and the pharmacokinetic parameters are summarized in Tables 1, 2. Compared with free-pirfenidone, PSLs could provide a higher blood concentration and longer half-life under both modes of administration. The drug profile in plasma and extended elimination half-life suggested that PSLs could improve the rapid elimination of pirfenidone *in vivo* and reduce fluctuations in plasma concentration, thereby reducing the risk of adverse reactions of pirfenidone. The plasma concentration of free-pirfenidone administered *via* the trachea was much lower than that of PSLs, which may have been because free-pirfenidone cannot penetrate the mucus of airways effectively compared with PSL, so only a small amount of pirfenidone was delivered to rats. Compared with intravenous injection, the peak time of PSLs *via* intratracheal administration was delayed, and the blood concentration was reduced. These phenomena may have been because administration to the pulmonary system increases the retention of pirfenidone in the lungs, suggesting that drug delivery to the pulmonary system has superior lung-targeting ability compared with that using intravenous injection. This result will be studied further in biodistribution studies and live imaging of small animals.

**TABLE 1:** Pharmacokinetic parameters of pulmonary administration of pirfenidone and PSLs in rats (4 mgkg^−1^) (*n* = 6).

Parameter	Pirfenidone	PSL
AUC_0-t_ (μg·h·mL^−1^)	0.29 ± 0.02	22.16 ± 8.54
AUC_0-∞_ (μg·h·mL^−1^)	0.31 ± 0.03	22.22 ± 9.37
T_1/2α_ (h)	0.022 ± 0.30	0.546 ± 0.02
T_1/2β_ (h)	1.826 ± 0.35	7.49 ± 0.67
T_max_ (h)	0.083	4.00 ± 0.47
C_max_ (ng/ml)	280.16 ± 120.32	5,300 ± 2,100

**TABLE 2:** Pharmacokinetic parameters of pirfenidone and PSLs in rats injected via the tail vein (4 mgkg^−1^) (*n* = 6).

Parameter	Pirfenidone	PSL
AUC_0-t_ (μg·h·mL^−1^)	19.45 ± 6.54	48.65 ± 20.16
AUC_0-∞_ (μg·h·mL^−1^)	20.25 ± 7.32	49.51 ± 23.41
T_1/2α_ (h)	0.023 ± 0.20	0.423 ± 0.05
T_1/2β_ (h)	1.624 ± 0.67	5.851 ± 0.45
T_max_ (h)	0.083	0.083
C_max_ (ng/ml)	9,720 ± 3,780	10,080 ± 5,648

#### 3.4.2 Biodistribution Study

**FIGURE 7:**
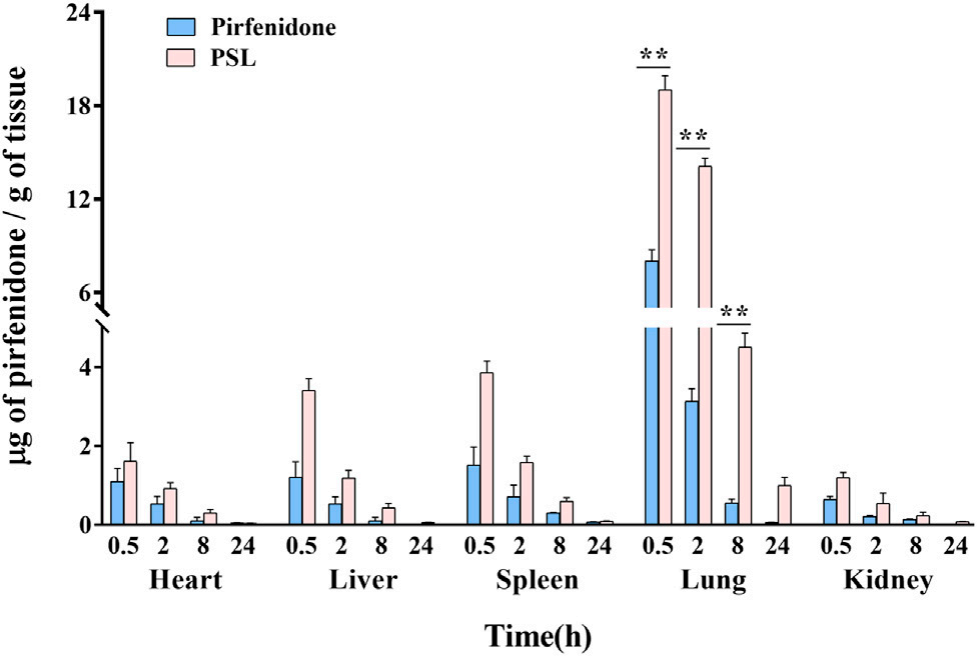
Pirfenidone level in the heart, liver, spleen, lung and kidney of rats with pulmonary fibrosis after administration to the pulmonary system (4 mgkg^−1^) (*n* = 3) (**p < 0.01).

To compare the distribution of PSLs and free-pirfenidone in the lungs by intratracheal administration, rats were sacrificed at certain intervals after administration, and tissue homogenates were collected to determine the pirfenidone concentration. The pirfenidone concentration in rats treated with PSLs was much higher in all organs compared with that upon treatment with pirfenidone solution (Figure 7). This result indicated that PSLs could penetrate the mucus layer of airways effectively and increase the amount of pirfenidone entering the lungs and body, which provides the feasibility of reducing the drug dose. The pirfenidone concentration in PSLs was higher in the lungs compared with that in other organs, suggesting that administration to the pulmonary system could increase pirfenidone retention in the lungs and reduce its distribution in other organs. This result indicated that the selectivity of PSLs to the lung was improved by administration to the pulmonary system, so the dose could be reduced.

#### 3.4.3 *In Vivo* Targeting Study

**FIGURE 8:**
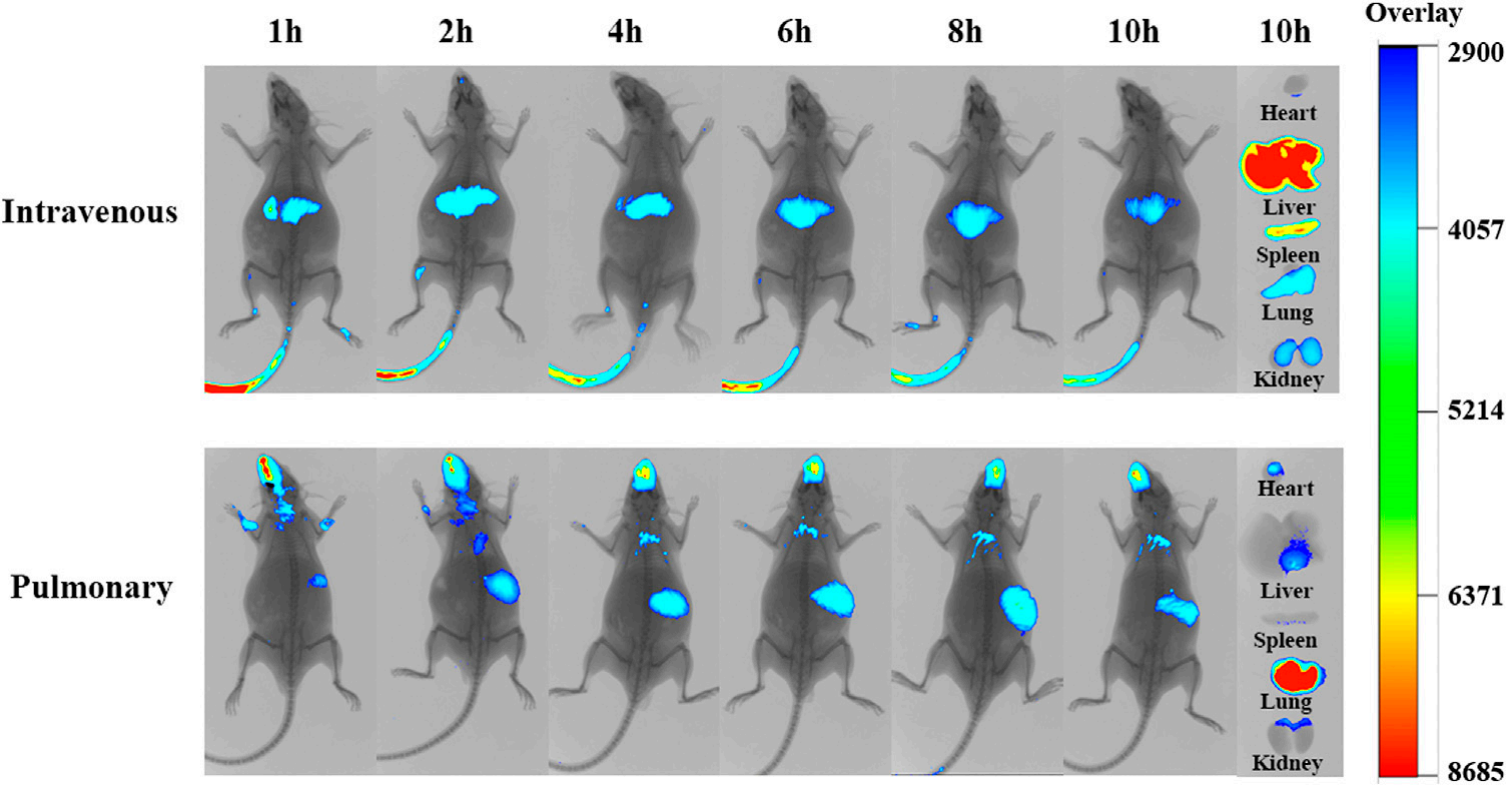
Fluorescence distribution in ICR mice after tail vein injection of DiR-PSL and pulmonary administration of DiR-PSL (0.25 mgkg^-1^) (*n* = 6).

We wished to compare the effect of the route of administration on the *in vivo* behavior of liposomes. An *in vivo* imaging study was undertaken in mice administered *via* tail-vein injection or intratracheal spray. After tail-vein injection, the fluorescence intensity *in vivo* was stronger in the liver and spleen, but weaker in the lung (Figure 8). This distribution was because liposomes were captured readily by the reticuloendothelial system in the blood circulation. However, fluorescence accumulation in the lungs was greater and the residence time was longer in mice administered the drug to the lungs. Hence, administration to the lungs with a local targeting effect was an optimal strategy in treatment of pulmonary fibrosis compared with intravenous injection.

#### 3.4.4 Study of Anti-Fibrosis Efficacy

**FIGURE 9:**
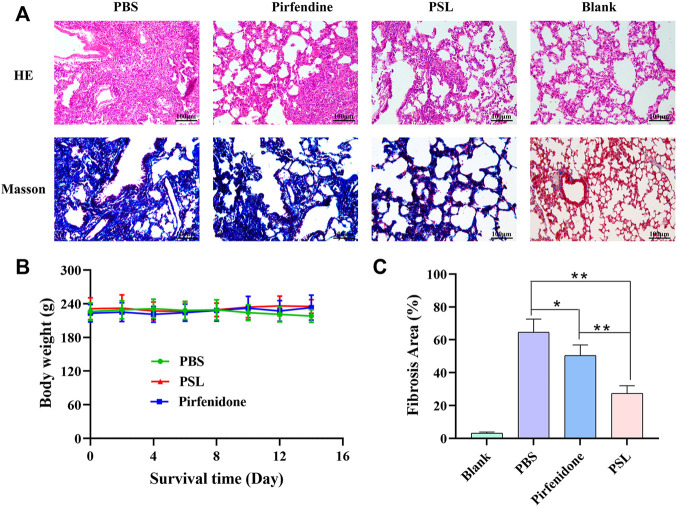
Results of H&E staining and Masson staining in rats with and without pulmonary fibrosis **(A)**. Body weight changes in mice from each group **(B)**. Quantification of fibrosis area in rats (*n* = 4) (**p < 0.01, *p < 0.05) **(C)**.

To evaluate the anti-fibrotic effect of PSLs, healthy rats were used as controls and the lung-tissue sections of rats with pulmonary fibrosis were observed after PSLs administration (Figure 9A). Compared with healthy rats, the alveoli of the PBS group were atrophied and collapsed, the lungs became solid, and many inflammatory cells infiltrated the tissues in the H&E-stained section. Pirfenidone solution had a therapeutic effect on fibrosis, but the alveoli were more fused and the number of blue-stained fibrotic cells was greater and accompanied by alveolar collapse and infiltration of inflammatory cells. However, only a small level of alveolar atrophy, infiltration of few inflammatory cells, and few blue-stained fibrotic cells were observed in the PSL group, indicating that PSLs administered to the lungs could hinder pulmonary fibrosis. The quantitative results of the fibrotic area of Masson-stained sections confirmed this conclusion, and PSL administration to the lungs could inhibit fibrosis progression significantly (Figure 9C). In addition, during the treatment period of 14 days, the fluctuation in the bodyweight of rats in each group was small, which suggested the favorable biosafety of PSLs (Figure 9B). These results indicated that, compared with pirfenidone solution, the PSLs we developed had a significant inhibitory effect on IPF, and may be a promising DDS for IPF therapy.

## 4 Conclusion

We developed pirfenidone PSLs for administration to the pulmonary system to overcome the mucus layer of airways and improve the shortcomings of traditional administration of pirfenidone. In an experiment on ASM penetration, pirfenidone PSLs modified with a certain density of PEG2000 showed obvious mucus-penetration ability and could be used for drug delivery *via* the lungs. Comparing the particle size and encapsulation efficiency of PSLs before and after atomization, the results show that the PSLs developed by us could remain relatively stable and could be used for subsequent experiments. Pharmacokinetic and biodistribution studies revealed that PSLs administered to the lungs prolonged the elimination time of pirfenidone and enabled significant retention in the lungs. In a model of pulmonary fibrosis in mice, PSLs, through lung administration, increased the lung-targeting of the drug compared with that with intravenous injection. Staining results suggested that PSLs significantly increased the ability of pirfenidone to inhibit the progression of pulmonary fibrosis in rats. Therefore, PSL administered to the lungs is a potential strategy for the treatment of IPF with improved lung targeting and drug dose reduction.

## Data Availability

The original contributions presented in the study are included in the article/Supplementary Material, further inquiries can be directed to the corresponding authors.
